# Thymol improves salinity tolerance of tobacco by increasing the sodium ion efflux and enhancing the content of nitric oxide and glutathione

**DOI:** 10.1186/s12870-021-03395-7

**Published:** 2022-01-13

**Authors:** Liang Xu, Jia-Qian Song, Yue-Lin Wang, Xiao-Han Liu, Xue-Li Li, Bo Zhang, Ai-Jie Li, Xie-Feng Ye, Jing Wang, Peng Wang

**Affiliations:** 1 College of Tobacco Science, Henan Agricultural University, Zhengzhou, 450002 China; 2 Guangdong Shaoguan Tobacco Recuring Co., LTD., Shaoguan, 512000 China; 3 China Tobacco Corporation Staff Training College, Zhengzhou, 450008 China; 4 Joint Center for Biomedical Innovation, Henan University, Kaifeng, 475000 China; 5 Wuhan Cigarette Factory of Hubei China Tobacco Industry Limited Liability Company, Wuhan, 430051 China

**Keywords:** Thymol, Tobacco, NO, GSH, Na^+^ efflux

## Abstract

**Background and objective:**

Salt stress is one of the most important abiotic stresses affecting the yield and quality of tobacco (*Nicotiana tabacum*). Thymol (a natural medicine) has been widely used in medical research because of its antibacterial and anti-inflammatory activities. However, the influence of thymol on the root growth of tobacco is not fully elucidated. In this study, the regulatory effects of different concentrations of thymol were investigated.

**Methodology:**

Here, histochemical staining and biochemical methods, non-invasive micro-test technology (NMT), and qPCR assay were performed to investigate the effect of thymol and mechanism of it improving salinity tolerance in tobacco seedlings.

**Results:**

In this study, our results showed that thymol rescued root growth from salt stress by ameliorating ROS accumulation, lipid peroxidation, and cell death. Furthermore, thymol enhanced contents of NO and GSH to repress ROS accumulation, further protecting the stability of the cell membrane. And, thymol improved Na^+^ efflux and the expression of *SOS1*, *HKT1*, and *NHX1*, thus protecting the stability of Na^+^ and K^+^.

**Conclusion:**

Our study confirmed the protecting effect of thymol in tobacco under salt stress, and we also identified the mechanism of it, involving dynamic regulation of antioxidant system and the maintenance of Na^+^ homeostasis. It can be a new method to improve salinity tolerance in plants.

**Supplementary Information:**

The online version contains supplementary material available at 10.1186/s12870-021-03395-7.

## Background

In recent years, soil salinization caused by natural and human factors has become a worldwide agroecological problem and continues to affect soil resources [1, 2]. More than one-fifth of the world’s arable land is currently under the threat of salt stress, which is a major challenge for plant growth [3, 4].

Salt stress affects the plant growth [5], with high salinity resulting in a decrease in the germination rate of seeds, inhibition on the growth of primary root growth, decrease in the number of lateral roots, and withering and yellowing of leaves [6]. Salinity also accelerates chlorophyll decomposition and decreases photosynthesis. In addition, salt stress causes osmotic stress, ion toxicity, and oxidative stress in plants, which damaging cellular components such as membrane lipids, proteins, and nucleic acids and causing metabolic dysfunction [7, 8]. As a result, salinity stress decreases the yield and the quality of crops always decrease upon salinity stress. Under salt stress, plants have evolved sophisticated mechanisms to cope with salinity stress, including selective ion uptake/exclusion, compartmentalization of toxic ions, synthesis of compatible products, adjustment of photosynthetic and energy metabolism, accumulation of antioxidative enzymes, regulation of hormones, and modification of cell structure [3, 5].

Preventing soil salinization and repairing saline soil may be the most fundamental approach to solving the salt stress problem; however, it has some limitations, such as difficult management, long period of application, and high cost. Therefore, it is important to find cheap, safe, effective, and practical regulators to alleviate salt stress. Exogenous regulation of plant salt tolerance is a kind of potential alternative. Presently, most of the exogenous substances are hormones, growth regulators, and signal substances. Therefore, it is essential to develop novel regulators with potential in field applications.

Thymol [5-methyl-2-(1-methylethyl) phenol] is an essential plant oil; it is a monoterpene phenol that is easily soluble in organic solvents. Thymol is widely used in medical research because of its relatively low price and low potential toxicity and risk. In addition, thymol has good antibacterial and anti-inflammatory activities, and can protect mouse livers by inhibiting lipid peroxidation [9]. In the food industry, thymol can be used as an antioxidant and food additive to maintain the quality of fresh food [10–12]. In addition, many studies have found that thymol modulates intrinsic plant physiology against Cd (cadmium) stress [13] and rice tolerance to salinity stress [14].

Thymol may improve tobacco tolerance to salinity stress because of the oxidation resistance and protective effect against Cd. In this study, we observed the phenotype of tobacco roots and measured ROS accumulation, NO and GSH content, and Na^+^/K^+^ transportation. This study reveals the mechanisms and protective effects of thymol and provides a reference for applying thymol in tobacco against salt stress.

## Results

### Thymol mitigates Salt-induced inhibition of tobacco root growth

**Fig. 1:**
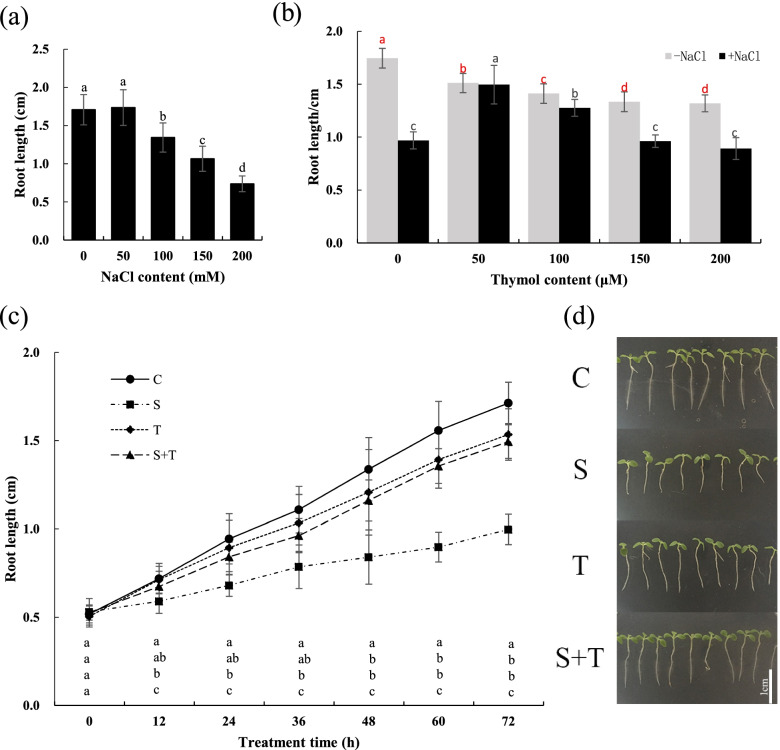
The effect of NaCl and thymol on the growth of tobacco seedlings. (**a**) The 6-day-old tobacco seedlings were transferred to plates containing 0–200 mM NaCl to measure root length after 72 h. (**b**) The seedlings were treated with 0–200 μM thymol and 150 mM NaCl for 72 h before measuring root length. (**c**) Tobacco seedlings were treated with water (C), 150 mM NaCl (S), 50 μM thymol (T) or the mixture of both (S + T) separately for 72 h. The root length was measured each 12 h. (**d**) The seedlings were photographed after 72-h treatment. Different letters indicate significant differences between the treatments (*p* < 0.05, ANOVA, LSD)

To characterize the effect of salt on the growth of tobacco seedlings, 6-day-old seedlings were treated with 0–200 mM NaCl for 72 h and the root length was measured. We found that 150 mM NaCl significantly inhibited root growth and reduced root length by 50% compared with the control (Fig. 1a). Then, the tobacco seedlings were allowed to grow in MS (Murashige & Skoog) medium containing 150 mM NaCl and different concentrations of thymol. Root length was slightly affected by thymol treatment alone (Fig. 1b). However, compared to NaCl treatment alone, the addition of thymol at 50 and 100 mM remarkably increased root length (Fig. 1b). Finally, using a time-course experiment, we cultured the seedlings in MS medium containing water (C), 150 mM NaCl (S), 50 μM thymol (T), or a mixture of these (S + T). The results indicated that roots treated with S + T showed a higher root growth rate than those treated with S (Fig. 1c and d), further supporting that thymol mitigated salt-induced inhibition of tobacco root growth.

### Thymol suppresses ROS accumulation in roots and leaves under Salt stress

**Fig. 2:**
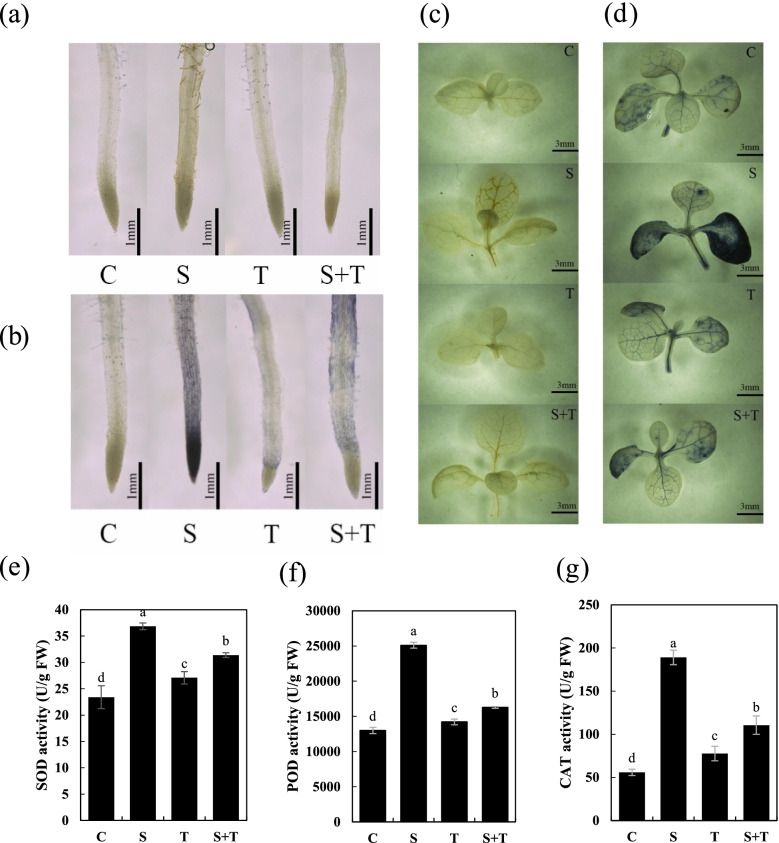
The effect of thymol on the accumulation of ROS in tobacco leaves and roots under salt stress. Tobacco seedlings were treated with water (C), 150 mM NaCl (S), 50 μM thymol (T) or the mixture of both (S + T) separately for 72 h. The roots were stained with DAB (**a**) or NBT (**b**) for 20 min to indicate H_2_O_2_ and ${\mathrm{O}}_2^{\bullet -}$ content, respectively. The seedlings were stained with DAB (**c**) or NBT (**d**) for 6 h to indicate H_2_O_2_ and ${\mathrm{O}}_2^{\bullet -}$ content in leaves. The activities of (**e**) SOD, (f) POD and (**g**) CAT. Different lowercase letters in e-g, indicated that the mean values of three replicates were significantly different between the treatments (*P* < 0.05, ANOVA, LSD)

**Fig. 3:**
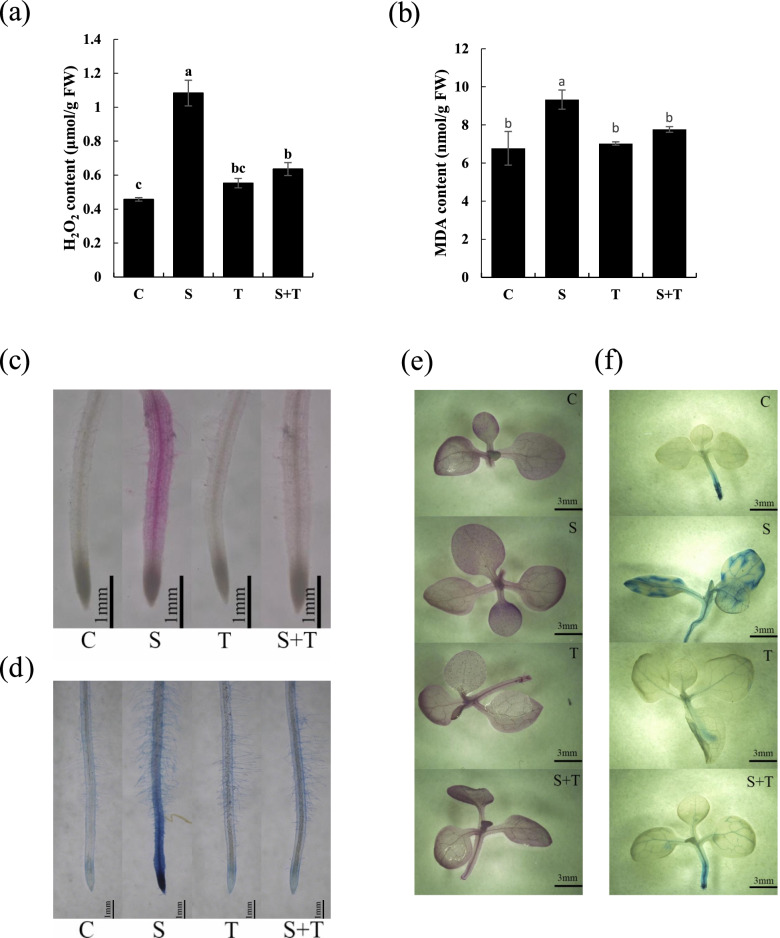
H_2_O_2_ and MDA content in tobacco seedlings and the effect of thymol on the lipid peroxidation and cell death in tobacco leaves and roots under salt stress. Tobacco seedlings were treated with water (C), 150 mM NaCl (S), 50 μM thymol (T) or a mixture of both (S + T) separately for 72 h. H_2_O_2_ content (**a**) and MDA content (**b**) in tobacco seedlings were detected using the kit. (**c**) The roots were stained with Shiff’s reagent for 20 min to indicate lipid peroxidation. (**d**) The roots were stained with trypan blue for 20 min to indicate cell death. (**e**) The leaves were supplied with Shiff’s reagent for 6 h to indicate lipid peroxidation. (**f**) Leaves were supplied with trypan blue for 6 h to indicate cell death in the leaves. Different lowercase letters in a-b indicate that the mean values of three replicates were significantly different between the treatments (*P* < 0.05, ANOVA, LSD)

Reactive oxygen species accumulation induced by salt stress is widely recognized as an important cause of damage to plants [15]. We tested whether the H_2_O_2_ content in tobacco seedlings was regulated by thymol. Treatment with 150 mM NaCl resulted in a significant (*P* < 0.05) increase in H_2_O_2_ content; seedlings under S + T treatment had much lower H_2_O_2_ content, similar to the control group (Fig. 3 a). Then, endogenous H_2_O_2_ and ${\mathrm{O}}_2^{\bullet -}$ levels were evaluated in situ in tobacco roots and leaves using DAB and NBT, respectively. The staining of seedlings grown on 150 mM NaCl was darker than that of the control; seedlings treated with S + T showed minor staining (Fig. 2a, b, c, and d). These results suggest that thymol decreases salt-induced ROS accumulation in tobacco seedlings.

ROS can act as a signal to activate the antioxidative system under stress conditions in plants. Salt stress-induced ROS accumulation is accompanied by the enhancement of the activities of three antioxidative enzymes: SOD, POD, and CAT (Fig. 2e, f, and g). However, the addition of thymol decreased the activity of these enzymes in the salt-treated seedlings. This may be due to decreased endogenous ROS levels upon thymol application.

### Thymol ameliorated salt-induced lipid peroxidation and cell death in roots and leaves

MDA is the main product of lipid peroxidation. The increased MDA content and H_2_O_2_ content in tobacco seedlings showed a strong response to salt stress, which was repressed by applying thymol (Fig. 3a and b). Schiff’s reagent was used to measure lipid peroxidation in situ. Under salt stress, the roots and leaves were stained pink, while those treated with S + T were stained lighter (Fig. 3c and e). Trypan blue was used to test cell death in tobacco roots and leaves. The results showed that root tips and leaves under salt stress showed extensive blue staining, while other seedlings were slightly stained (Fig. 3d and f). These results suggest that thymol could attenuate lipid oxidation and cell death in tobacco seedlings under salt stress.

### Thymol increased the NO and GSH content in tobacco seedlings under salt stress

**Fig. 4:**
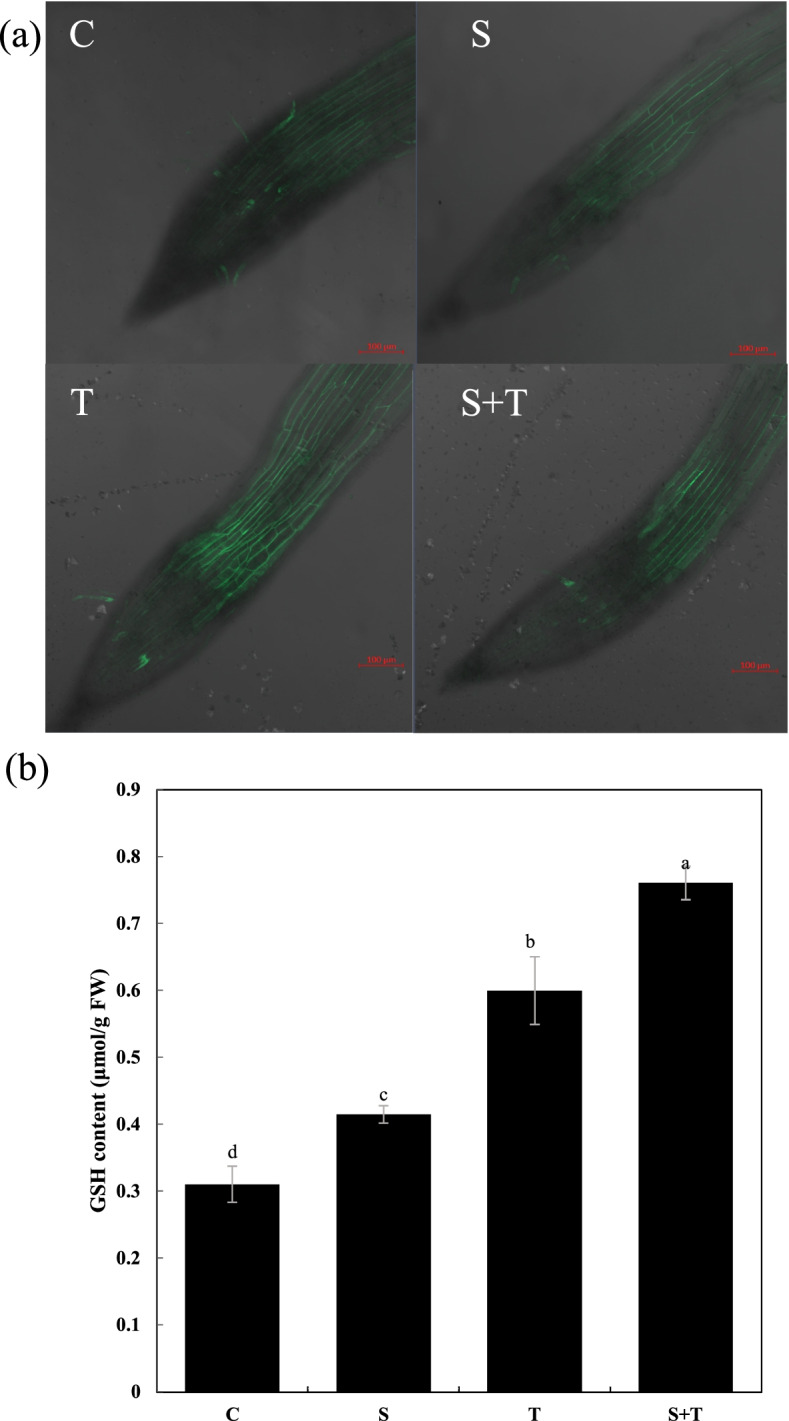
Effect of thymol on NO and antioxidant system in tobacco seedlings under salt stress. DAF-FM DA fluorescence indicates total NO level (**a**). (**b**) GSH content. Different lowercase letters in b, indicated that the mean values of three replicates were significantly different between the treatments (*P* < 0.05, ANOVA, LSD)

NO is an important defensive signaling molecule that helps combat salt stress [16]. Endogenous NO in tobacco roots was detected in situ using a specific fluorescent probe DAF-FM DA. In this study, we found that thymol enhanced endogenous NO levels in roots under salt stress (Fig. 4a). Furthermore, GSH is a major antioxidant in plants, and the addition of thymol significantly increased the GSH content in tobacco seedlings in the presence or absence of NaCl (Fig. 4b). These results suggest that NO and GSH may be involved in thymol-facilitated salt tolerance in tobacco seedlings.

### Thymol mediated Na^+^/K^+^ transportation in tobacco seedlings under salt stress

**Fig. 5:**
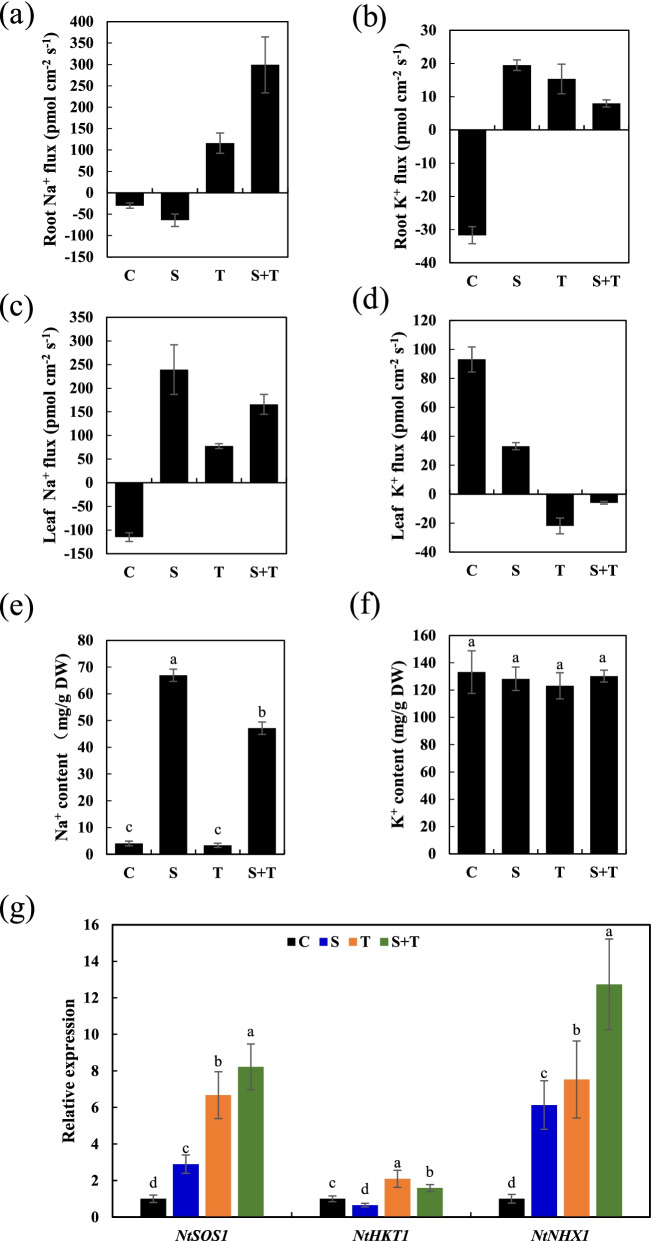
Effect of thymol on Na^+^ and K^+^ content and the expression of Na^+^ transporter genes in tobacco seedlings under salt stress. Na^+^ and K^+^ flux in tobacco roots and leaves (**a**-**d**). Na^+^ and K^+^ content in tobacco seedlings (**e** and **f**). Relative expression level of *NtSOS1*, *NtHKT1* and *NtNHX1* (**g**). Different lowercase letters indicated that the mean values of three replicates were significantly different between the treatments (*P* < 0.05, ANOVA, LSD). The expression levels of *NtSOS1*, *NtSOS1* and *NtSOS1* in control were defined as “1”. Data are means ± SE (*n* = 3)

Maintaining a balanced cytosolic Na^+^/K^+^ ratio has become a key salinity tolerance mechanism. Achieving this homeostatic balance requires the modulation of Na^+^ and K^+^ transporters and/or channels. The Na^+^ and K^+^ fluxes in the root tips and leaves were measured using non-invasive micro-test technology (NMT). We found that Na^+^ influx in the root tip was induced by salt while thymol caused Na^+^ efflux (Fig. 5a), suggesting that the absorption of Na^+^ was inhibited by thymol in the root tip. In contrast to that in the root tip, Na^+^ efflux was observed in leaves treated with salt or thymol (Fig. 5c). In addition, we measured Na^+^ content in seedlings and the expression of known Na^+^ transporter genes, including *NtSOS1*, *NtHKT1*, and *NtNHX1*. Thymol decreased Na^+^ content in seedlings under salt stress (Fig. 5e). In addition, thymol enhanced the expression levels of *NtSOS1*, *NtHKT1*, and *NtNHX1* as compared to those in the salt treatment (Fig. 5g). These results suggest that thymol modulates Na^+^ transporters to maintain K^+^/Na^+^ homeostasis in tobacco seedlings under salt stress.

## Discussion

Thymol has drawn great attention from scientific researchers because of its antioxidant properties [17]. In this study, we found that thymol enhanced plant tolerance by increasing NO and GSH content, and modulating Na^+^/K^+^ homeostasis in tobacco seedlings.

Increased ROS formation was observed in plants in response to both osmotic and ionic stresses associated with soil salinity, further resulting in oxidative stress and cell damage [18]. ROS (H_2_O_2_ and superoxide anion), can directly damage most cellular macromolecules and cause irreversible damage [8]. In contrast, ROS have also been proposed to act as signaling mediators of plant salinity tolerance. Plants scavenge excess ROS through the antioxidant system to maintain cell homeostasis and reduce the harm caused by oxidative stress.

In the present study, we found that the growth of tobacco seedlings was strongly affected by salt stress. Salt stress significantly inhibited the root growth of tobacco seedlings, which was attenuated by thymol treatment (Fig. 1). Under salt stress, H_2_O_2_ and ${\mathrm{O}}_2^{\bullet -}$ both in leaves and roots were decreased by thymol, suggesting that thymol effectively prevented the over-accumulation of ROS in tobacco leaves and roots (Fig. 2). Furthermore, thymol led to a decrease in MDA content and the lighter staining of Schiff’s reagent in tobacco seedlings under salt stress, suggesting that lipid peroxidation and oxidative injury caused by salt stress were ameliorated by thymol (Fig. 3). ROS directly affects normal cellular functioning and leads to cell death [19]. Therefore, our experiments proposed that the decrease in ROS accumulation caused by thymol resulted in the mitigation of lipid peroxidation and cell death in salt-treated tobacco seedlings.

Plants scavenge excess ROS through the antioxidant system to maintain cell homeostasis and reduce the harm caused by oxidative stress. Antioxidant systems include enzymes such as SOD, POD, and CAT and non-enzymatic antioxidants, such as glutathione, ascorbic acid, and carotenoids, which are important mechanisms for plants to resist salt stress [20]. Under salt stress, the activities of SOD, POD, and CAT were increased to scavenge the over-accumulation of ROS. Furthermore, it was significantly increased when treated with thymol, suggesting that thymol is an active antioxidant system that modulates ROS homeostasis (Fig. 2).

NO, an essential messenger that exists widely in plants, regulates multiple plant growth and development processes. NO also modulates plant responses to various abiotic stresses, including salt, drought, and heavy metals. NO helps eliminate ROS through two pathways. First, NO can regulate ROS levels [21–23] by regulating ${\mathrm{O}}_2^{\bullet -}$ and H_2_O_2_ production [21]. Second, nitroso glutathione (GSNO), generated via the reversible reaction of NO and GSH, is an endogenous regulator of NO and GSH homeostasis [24]. GSH reacts directly with ROS species [25], and participates in the ASA-GSH cycle to scavenge ROS [26, 27]. In this study, we found that thymol enhanced endogenous NO and GSH content in tobacco seedlings under salt stress. This may explain the decreased ROS content in the salt-treated seedlings in the presence of thymol. However, further studies are needed to determine whether thymol modulates GSNO to maintain the homeostasis of NO, GSH, and ROS upon salt stress.

**Fig. 6:**
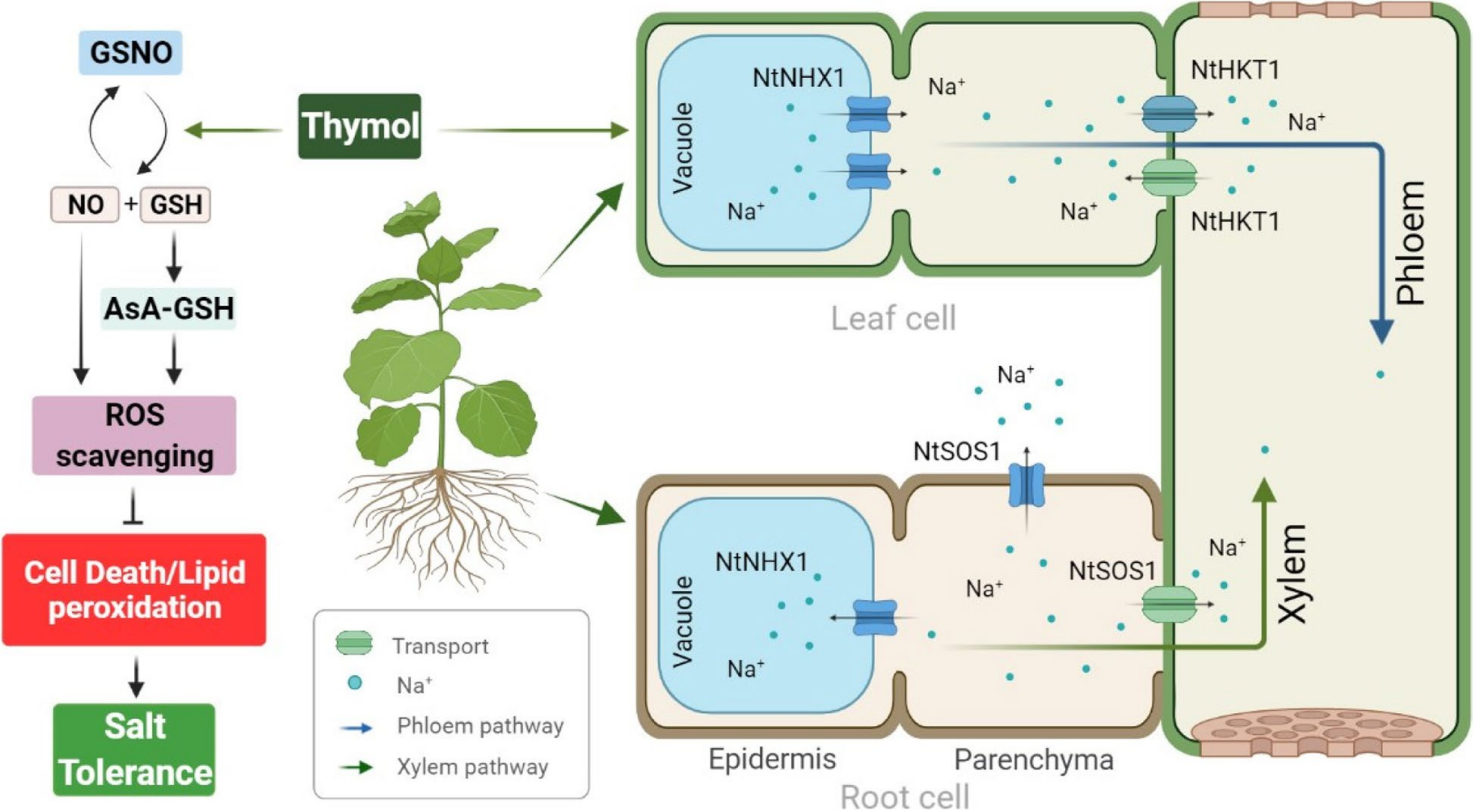
Schematic model for thymol-induced salt tolerance in tobacco seedlings. Thymol deploys two strategies to increase the salt tolerance of tobacco seedlings. First, thymol promotes the scavenging of ROS by promoting the decomposition of GSNO and increasing the content of NO and GSH. Second, thymol activates *NtNHX1* to stimulate vacuole retention of Na^+^, *NtSOS1* to promote Na^+^ rejection, and *NtHKT1* to promote the transfer of Na^+^ to xylem and phloem, thus helping to reduce the level of Na^+^ in leaves and roots. (Created with BioRender.com)

Ionic stress, caused by excess Na^+^ accumulation, is an important component of salt stress. To relieve Na^+^ toxicity, some strategies have been developed to decrease Na^+^ content in the cytosol of plant cells, including suppression of Na^+^ uptake and enhancement of Na^+^ compartmentalization [28]. The plasmalemma Na^+^/H^+^ antiporter, SOS1, transports Na^+^ out of the cell; high-affinity K^+^ transporter, HKT, transports Na^+^ from stems to xylem; sodium-hydrogen exchanger, NHX, transports Na^+^ into the vacuole [29, 30]. In this study, Na^+^ influx was observed in roots treated with salt, which was repressed by the addition of thymol due to the activation of *NtSOS1* by thymol. Thymol also induced the expression of *NtHKT1*, which is responsible for the tissue distribution of Na^+^, which may also contribute to the decrease in total Na^+^ content in roots. K^+^ in tobacco seedlings was not significantly affected. Thus, thymol seems to maintain Na^+^ / K^+^ balance, likely through the regulation of Na^+^ transportation. In addition, *NtNHX1* was induced by thymol, which may help root cells compartmentalize Na^+^ inside the vacuole to avoid Na^+^ toxicity in the cytosol. However, the subcellular distribution of Na^+^ in thymol-treated tobacco seedlings requires further investigation (Fig. 6).

## Conclusion

In this study, histochemical detection, histochemical staining, qPCR assay, and NMT were performed to demonstrate the protective effect of thymol in tobacco seedlings against salt stress. Our results indicated that the application of thymol increased SOD, POD, and CAT activities for scavenging ${\mathrm{O}}_2^{\bullet -}$ and H_2_O_2_; hence, the stability of the membrane system was maintained, and cell death decreased in tobacco seedlings. Additionally, it increased NO and GSH contents by promoting the decomposition of GSNO to scavenge the excessive accumulation of ROS. Furthermore, thymol activated *NtNHX1*, *NtSOS1*, and *NtHKT1*, which caused the retention of Na^+^ in vacuole, promotion of Na^+^ rejection, and promotion of the transfer of Na^+^ to xylem and phloem, respectively, thus decreasing the Na^+^ content to relieve ion toxicity in tobacco under salt stress. The detailed molecular mechanisms are still elusive, but these results provide a certain understanding of the physiological functions of thymol regarding to salt tolerance.

## Methods

### Plant culture, treatment, and chemicals

The tobacco seeds (K326) were washed ten times with distilled water and sown on the surface of the culture medium in a petri dish after being disinfected with 0.2% potassium permanganate solution for 30 min. (The tobacco seeds were provided by Yuxi Zhong Yan Seed Co., Ltd) They were cultivated for 6–7 days in a plant growth cabinet with a light intensity of 5000 lx, air relative humidity of 50%, photoperiod of 12 h, and temperature of 26 °C. Then, seedlings with a root length of 0.5 cm were selected and transplanted into a new Petri dish containing NaCl and thymol alone or in combination for 72 h. NaCl (0–200 mM) was added to the MS medium before sterilization. Thymol (0 - 200 μM) was mixed into 10,000 times mother liquor with alcohol, and then filter sterilized mother liquor was added to sterilized MS medium. The root length was measured every 12 h. After 72 h of incubation, the plants were harvested for histochemical, physiological, and biochemical analyses.

### Histochemical detection

The DAB, NBT, Schiff’s reagent and trypan blue were used for the detection of H_2_O_2_, ${\mathrm{O}}_2^{\bullet -}$, lipid peroxidation and cell death in roots and leaves, according to our previous publication [13].

To detect indicators in roots, 6-day-old seedlings treated for 72 h were transferred to different solutions at 25 °C for 20 min under a light. Then, the roots were washed with deionized water until apical discoloration was observed, and the root tips were photographed under a stereoscopic microscope (SteREO Discovery.V8, ZEISS, Oberkochen, Germany).

The seedlings were excised at the base of the stem and stained with DAB, NBT, trypan blue, or Schiff’s reagent for 6 h to indicate H_2_O_2_, ${\mathrm{O}}_2^{\bullet -}$ content, lipid peroxidation, and cell death in leaves. The leaves were then washed and boiled with 95% alcohol for 20 min and photographed using a stereomicroscope.

### Determination of H_2_O_2_, MDA, GSH, NO content and enzymatic activity

The harvested plants were collected and ground to powder in a mortar containing liquid nitrogen by spectrophotometric method to detect H_2_O_2_ (hydrogen peroxide), malondialdehyde (MDA), reduced glutathione (GSH), peroxidase (POD), superoxide dismutase (SOD), and catalase (CAT). All kits were manufactured by the Suzhou Comin Biotechnology Co., Ltd., China. The subsequent measurement and data analysis were conducted following the operational manuals of the kits. The amount of NO was analyzed using DAF-FM DA staining and confocal laser scanning microscopy, and the kits were manufactured by the Beyotime Biotechnology Co. Ltd., China.

### K^+^ and Na^+^ flux assays

The 6-day-old seedlings treated for 12 h were used for K^+^ and Na^+^ determination. K^+^ and Na^+^ efflux in tobacco leaves and roots were detected using non-invasive micro-test technology (NMT; Younger LLC) as previously described [31].

### Gene expression analysis

Total RNA was isolated from tobacco seedlings using an RNA extraction kit (Takara, Japan) and reverse-transcribed using the Prime Script TM RT-PCR Kit (Takara, Japan), following the manufacturer’s instructions. The qPCR assay was performed on a Quant Studio 5 Real-Time PCR system (Applied Biosystems, CA, USA) using SYBR Green PCR Master Mix (Takara, Japan). The PCR conditions were 95 °C for 15 s and 60 °C for 1 min for 40 cycles. Relative gene expression of the *NtSOS1*, *NtHKT1* and *NtNHX1* transcripts was calculated using the 2^-△△CT^ method. All qPCR analyses were performed using three independent biological replicates.

The sequences of these genes were retrieved from the National Center for Biotechnology Information (https://www.ncbi.nlm.nih.gov/) for primer design. Primers for each gene used in this study are presented in Table S1.

### Statistical analysis

The mean ± standard deviation (SD) of ten replications was used to present the data. After ANOVA, Student’s t-test was used to identify significant differences (*P* < 0.05) among treatment means. In addition, LSD (least significant difference test) was used to test for significant differences (*P* < 0.05) among the different treatments in one experiment.

## Supplementary Information

**Additional file 1.** Primers used for qPCR (Table S1).

## Data Availability

All of the data and materials supporting our research findings are contained in the Methods section of the manuscript. The details are provided in the attached supplementary files.

## Abbreviations

ROS: Reactive oxygen species
NO: Nitric oxide
GSH: Glutathione
MS: Murashige & Skoog
DAB: Diaminobenzidine
NBT: Nitro blue tetrazolium chloride
H_2_O_2_: Hydrogen peroxide
MDA: Malondialdehyde
POD: Peroxidase
SOD: Superoxide dismutase
CAT: catalase
DAF-FM DA: 3-Amino,4-aminomethyl-2′,7′-difluorescein, diacetate
GSNO: S-nitrosoglutathione
ASA: Ascorbic acid
